# Challenges and opportunities for the early identification of ADHD across multiple levels of analysis – A reflection on Tobarra‐Sanchez et al. (2022)

**DOI:** 10.1002/jcv2.12101

**Published:** 2022-08-24

**Authors:** Meghan Miller

**Affiliations:** ^1^ Department of Psychiatry & Behavioral Sciences and MIND Institute University of California Davis California USA

**Keywords:** ADHD, early identification, prediction

The number of publications examining early markers of ADHD is rapidly growing (see recent review and meta‐analysis by Shephard et al., 2022), each contributing important scientific information. Although some of the reported findings are also clinically informative, the chasm between these scientific findings and clinical translation remains large. This is, in part, due to approaches that prioritize individual early predictors rather than more comprehensive, multivariable examinations of early markers of ADHD which evaluate contributions of numerous predictors simultaneously. Moreover, many published studies fail to incorporate predictors spanning multiple levels of analysis, from biology to behavior.

As the first study to include a combination of genetic risk via ADHD polygenic risk scores (PRS) and clinical, developmental, and behavioral predictors acquired over the first 30 months of life in relation to later childhood ADHD, Tobarra‐Sanchez et al. (2022) have taken an important step toward better understanding early predictors of ADHD. Making use of the Avon Longitudinal Study of Parents and Children (ALSPAC), a large and well‐characterized UK population‐based birth cohort, the authors examined a sequence of univariable models culminating in a multivariable model aimed at predicting later ADHD outcomes. Ultimately, they found that the ADHD PRS, temperament‐based activity level at 2 years of age, lower parent‐rated fine motor skills at 18 months of age, and lower parent‐rated vocabulary at 24 months of age predicted later dimensionally measured ADHD *symptoms*, while ADHD PRS and temperament‐based activity level predicted DSM‐IV ADHD *diagnosis*.

The potential implications of this work for future research are numerous. For example, it highlights the pressing need for prospective studies from birth specifically designed to address questions about early markers of ADHD (despite known limitations of such designs). Large birth cohort studies provide invaluable information but, due to their nature, may rely more heavily on parent‐report metrics versus direct assessment and deep phenotyping, along with variable batteries across time. When used to address research questions that they were not necessarily intended to answer at the outset, some limitations are therefore imposed. In this case, data were acquired across multiple ages between 15 and 30 months and were not always simultaneously measured, likely resulting in a loss of developmental nuance. For example, hearing/vision concerns were assessed at 15 months, motor skills were assessed at 18 months, vocabulary and temperament were assessed at 24 months, and regulatory issues were evaluated at both 24 (feeding) and 30 (sleeping/crying) months; these data were pooled in multivariable models despite dramatic changes in development during this period. Indeed, development is particularly dynamic at such early ages, making it difficult to extrapolate precise developmental meaning or potential effects of developmental cascades. Prospective studies focused on early markers of ADHD will provide opportunities to build upon Tobarra‐Sanchez et al.’s (2022) findings by carefully tracking development of each of these identified domains. They will also more fully address questions related to heterotypic and homotypic continuity and, relatedly, whether there is variation in the composition of predictive multivariable models based on different ages and stages of development.

Equally important to what *did* predict ADHD outcomes, this study raises intriguing questions based upon what *did not* predict ADHD outcomes in the univariate models, or which *did* predict ADHD outcomes in univariate models but not once included in the multivariable model. This includes a range of pre‐ and perinatal variables (e.g., young maternal age at birth, preterm birth, intrauterine growth restriction, low APGAR score), as well as child‐level behavioral variables including temperament‐based distractibility and regulatory problems (sleeping, crying, feeding problems), and developmental variables (e.g., gross motor delay, grammar delay). This may suggest that downstream child‐level behavioral and developmental characteristics, in addition to genetic risk, are more influential than factors associated with the pre‐/perinatal periods. Alternatively, methodological decisions, particularly the approach of dichotomizing the majority of predictors in favor of clinical utility, could have resulted in a loss of nuance. Also informative to future investigations are the inter‐relations among predictors, particularly given the inclusion of the ADHD PRS. Notably, the ADHD PRS z‐score did not correlate with any variables *other than* those related to the core dimensions or diagnosis of ADHD (SDQ Hyperactivity, DAWBA ADHD, temperament‐based activity), with one exception (lower maternal age), suggesting some degree of specificity.

The incorporation of genetic risk into multivariable models investigating early markers of ADHD is a key advancement and aligns with widely accepted theories about the development of psychopathology. Despite this, there are some limitations to the use of PRS. These have been described elsewhere (Shaw, 2022), but a key concern relates to the fact that the majority of genome wide association studies (GWAS) have been conducted in European samples and are not globally representative. Because polygenic risk scores are based on ‘discovery’ GWAS samples, there are therefore questions about generalizability with which the field must contend (Bien et al., 2019). Indeed, the sample included in Tobarra‐Sanchez et al.’s (2022) analyses excluded individuals on the basis of non‐European ancestry, and the vast majority of studies examining the ADHD PRS have been done in samples including primarily European ancestry (Ronald et al., 2021). While this may be scientifically justifiable based on the sample from which the risk scores were generated, if there is any hope for polygenic risk scores to provide future clinical utility, no matter how incremental, the field must make efforts to ensure that the growing literature base related to PRS in psychopathology is globally representative. At this stage, the ADHD PRS is useful for research purposes only (Ronald et al., 2021); these issues must be addressed before any future efforts toward clinical translation can be realized.

As the authors note, it will be critical for future research to focus on synergistic interactions between predictors as well as non‐linear interactions. Specificity is also an open question. For example, given that delayed motor and language development are well‐documented predictors of other neurodevelopmental conditions (Johnson et al., 2015), such patterns are clearly non‐specific, leading to questions about whether interventions addressing these domains may also have non‐specific, transdiagnostic effects. Whether precise patterns of interactions among predictors may display some specificity to outcomes, however, remains an area for exploration. Likewise, although the multivariable models were statistically significant, they accounted for a small proportion of variance in outcomes. Thus, practical and clinical significance remain elusive.

Although beyond the scope of the present paper, the work by Tobarra‐Sanchez et al. also (2022) emphasizes the need for the field to go beyond individual predictors and begin to develop and test prognostic diagnostic models (Larsson, 2021; Senior et al., 2021) which have direct translational implications. For example, prognostic models have the potential to contribute to the development of more accurate surveillance and screening measures and guidelines, which could help to identify which very young children are at greatest risk for developing ADHD years later, allowing for the application of potential pre‐emptive or supportive interventions. Such models are perhaps beyond the horizon as yet, but the work by Tobarra‐Sanchez et al. (2022) moves us closer.

## AUTHOR CONTRIBUTION

**Meghan Miller**: Conceptualization; Writing − original draft.

## CONFLICTS OF INTEREST

Meghan Miller reports no biomedical financial interests or potential conflicts of interest.

## Data Availability

Data sharing not applicable to this article as no datasets were generated or analyzed.
